# The NMR studies of CMP inhibition of polysialylation

**DOI:** 10.1080/14756366.2023.2248411

**Published:** 2023-08-24

**Authors:** Bo Lu, Si-Ming Liao, Xue-Hui Liu, Shi-Jie Liang, Jun Huang, Mei Lin, Li Meng, Qing-Yan Wang, Ri-Bo Huang, Guo-Ping Zhou

**Affiliations:** a National Engineering Research Center for Non-food Biorefinery, Guangxi Academy of Sciences, Nanning, Guangxi, China; b Institute of Biophysics, Chinese Academy of Sciences, Beijing, China; c Rocky Mount Life Sciences Institute, Rocky Mount, NC, USA

**Keywords:** Metastatic spread, polysialic acid, polysialyltransferase, polysialyltransferase domain, chemical shift perturbation

## Abstract

The overexpression of polysialic acid (polySia) on neural cell adhesion molecules (NCAM) promotes hypersialylation, and thus benefits cancer cell migration and invasion. It has been proposed that the binding between the polysialyltransferase domain (PSTD) and CMP-Sia needs to be inhibited in order to block the effects of hypersialylation. In this study, CMP was confirmed to be a competitive inhibitor of polysialyltransferases (polySTs) in the presence of CMP-Sia and triSia (oligosialic acid trimer) based on the interactional features between molecules. The further NMR analysis suggested that polysialylation could be partially inhibited when CMP-Sia and polySia co-exist in solution. In addition, an unexpecting finding is that CMP-Sia plays a role in reducing the gathering extent of polySia chains on the PSTD, and may benefit for the inhibition of polysialylation. The findings in this study may provide new insight into the optimal design of the drug and inhibitor for cancer treatment.

## Introduction

It has been known that NCAMs could be modulated by Polysialic acid (polySia), which is a distinctive glycan expressed on the surface of normal human neuronal cells. In mammalian cells, it is primarily linked posttranslationally to N-glycans on NCAM proteins^1–9^. These glycoproteins are also expressed on the surface of a number of human cancers where they may function as a metastatic factor^4^^,^^10–12^.

Peripheral adult organs express plySia-NCAM proteins less frequently than neural cells. However, some tumours of neural crest-origin, small cell lung cancers (SCLC), pancreatic cancers, neuroblastomas and malignant myelomas can re-express polySia-NCAM glycoproteins on their surface. This expression is often correlated with aggressive and invasive diseases with poor clinical outcomes^4^^,^^11–13^. Frequently overlooked, is that the ‘tumour associated’ polySia-NCAM proteins described above are the identical molecules that are of key importance in neurodevelopment/neurogenesis, neural plasticity, cognition, learning and memory^13–15^.

The synthesis of polySia can be catalysed by two polysialyltranseferases (polySTs), ST8Sia IV (PST) and ST8Sia II (STX)^7^^,^^8^^,^^10^^,^^15^. However, the overexpression of polySia on NCAM promotes hypersialylation, and benefits cancer cell migration and invasion. Several earlier studies in the area of polysialylation focused on identifying inhibitors of the polySTs to develop therapeutics to target metastatic tumours in humans^16^. The studies by Paulson and colleagues^17^ showed that polysialylation of NCAM could be inhibited by cytidine monophosphate (CMP). After that, Falconer, R.A. et al. postulated that the polysialyltransferases were a ‘new target’ for metastatic cancer^18^. In addition, it has also been known that CMP-sialic acid (CMP-Sia), the activated nucleotide sugar of N-acetylneuraminic acid (Neu5Ac; Sia)^19–25^, is required by the polySTs for the biosynthesis of polySia^9^^,^^26–28^. Accordingly, the synthesis of the polySia-NCAM protein may require the interactions of polyST-(CMP-Sia) and polyST-polySia in the lumen of the Golgi apparatus^13^^,^^29–32^.

In 2006, Nakata et al. identified that the polysialyltransferase domain (PSTD) is a polybasic motif of 32 amino acids in the polySTs^29^. PSTD has been proposed to be a functional domain in polySTs, and is essential for polysialylation of NCAM proteins based on the vitro experimental data and molecular modelling analysis^33–37^. The experimental results using Chinese human ovary cells suggested that the catalysis function of polySTs could be inhibited by CMP, and thus result in the decline of polySia expression on NCAM^38^. However, the molecular mechanism of the inhibition is still unclear.

So far, both 3D X-ray and NMR structures of the polySTs have not yet been reported, due to the existence of many hydrophobic residues in the polySTs, which are in the membrane environment^30^^,^^33^^,^^34^. Recently, the 3-D solution structure of the PSTD peptide, an active site in the ST8Sia IV, has been obtained based on our NMR studies^30^^,^^36^. Thus, a hypothesis about the interaction between the PSTD and the ligands may correspond to the interactions between the polyST and its ligands. This is an efficient research strategy and methodology for studying biological problems using biophysical and NMR structural biology, and the above hypothesis has been successfully tested by the recent NMR studies^30–32^.

More recent studies have shown that the NMR 3D structure of a synthesised 35-amino acid PSTD peptide is very similar to the predicted PSTD structure of the molecular model^30^^,^^36^. Thus, this NMR-derived 3-D structural model not only verifies that the predicted model is quite accurate but also shows that this synthetic peptide should be appropriate for NMR study of the interactions between the PSTD and the other ligands^36^. Using this NMR-derived structural model of the PSTD, we have found that CMP-Sia was preferentially bound to the short helix H1, and the region between H1 and the long helix H2. On the other hand, polySia was mainly bound to the H2 helix of the PSTD. In addition, the peak intensities of 20 residues in H2 helix were significantly decreased after polySia bound to the PSTD. This finding suggests a slow chemical exchange and the formation of the aggregates might appear in the binding region of polySia in the PSTD^30^^,^^36^.

To further investigate the molecular mechanisms of pharmacological inhibition of polyST by CMP and derivatives, the interactions of the PSTD-CMP were carried out to determine the molecular details of CMP inhibiting NCAM polysialylation.

## Materials and methods

### Material sources

The PSTD (246K-277R) should be a 32 amino acid sequence peptide from ST8Sia IV molecule. However, in order to obtain more accurate 3D structural information by NMR spectroscopy, one amino acid (245 L) and two amino acids (278 P and 279S) from ST8Sia IV sequence were added into the N- and C- terminals of PSTD, respectively^30^. Thus, a 35 amino acid sequence peptide sample containing PSTD was synthesised as follows: *245LKNKLKVRTAYPSLRLIHAVRGYWLTNKVPIKRPS279*’. In which, the PSTD sequence is labelled by underline. This intact peptide sample was chemically synthesised by automated solid-phase synthesis using the F-MOC-protection strategy and purified by HPLC (DG Peptide Company, Hangzhou, China). The molecular weight of this peptide sample was 4117.95 and its purity was 99.36%.

CMP and CMP-Sia were purchased from Santa Cruz Biotechnology. The molecular weights of CMP and CMP-Sia, are 323.2, and 614.50 g/mol, respectively.

### Circular dichroism (CD) Spectroscopy

The concentrations of the 35 amino acid-PSTD peptide and CMP in 20 mM phosphate buffer (pH 6.7) with 25% tetrafluoroethylene (TFE) were 8.0 uM and 400 uM, respectively. The measured and recorded methods of CD spectra are the same as the previous articles^30^^,^^37^.

### NMR Spectroscopy: Sample preparation

The 35 amino acid peptide containing the PSTD was prepared as described in reference^37^. Chemical shifts were referenced with respect to 2-dimethyl-2-silapentane-5-sulfonic acid (DSS) used as the internal standard. The ratio of CMP to the peptide was selected based on CD studies that indicated alterations in the secondary structure of the peptide in 25%TFE (*v*/*v*), 10% D_2_O (*v*/*v*), and 65% (*v*/*v*) 20 mM phosphate buffer (pH 6.7).

The concentrations of 35AA-PSTD peptide, CMP, CMP-Sia, triSiA (trimer of α-2,8-linked sialic acid DP3) and polySia (DP95) in the buffer were 2 mM, 1 mM, 1 mM, 1 mM, and 0.1 mM, respectively^30^^,^^37^ for all NMR experiments.

### NMR spectroscopic Methods

The NMR spectroscopic methods are the same as the previous paper^37^, and the chemical shift perturbation (CSP) of each amino acid in the PSTD was obtained using the formula:

(1) $$\text{CSP}={\left[\left(D^{2}{}_{\text{NH}}+{\left(D_{N}/5\right)}^{2}\right)/2\right]}^{1}{}^{/2}$$

where, D_N_ and D_NH_ represent the changes in ^15^N and ^1^H chemical shifts, respectively, upon ligand binding^39^.

## Results

### CD Data

**Figure 1.**
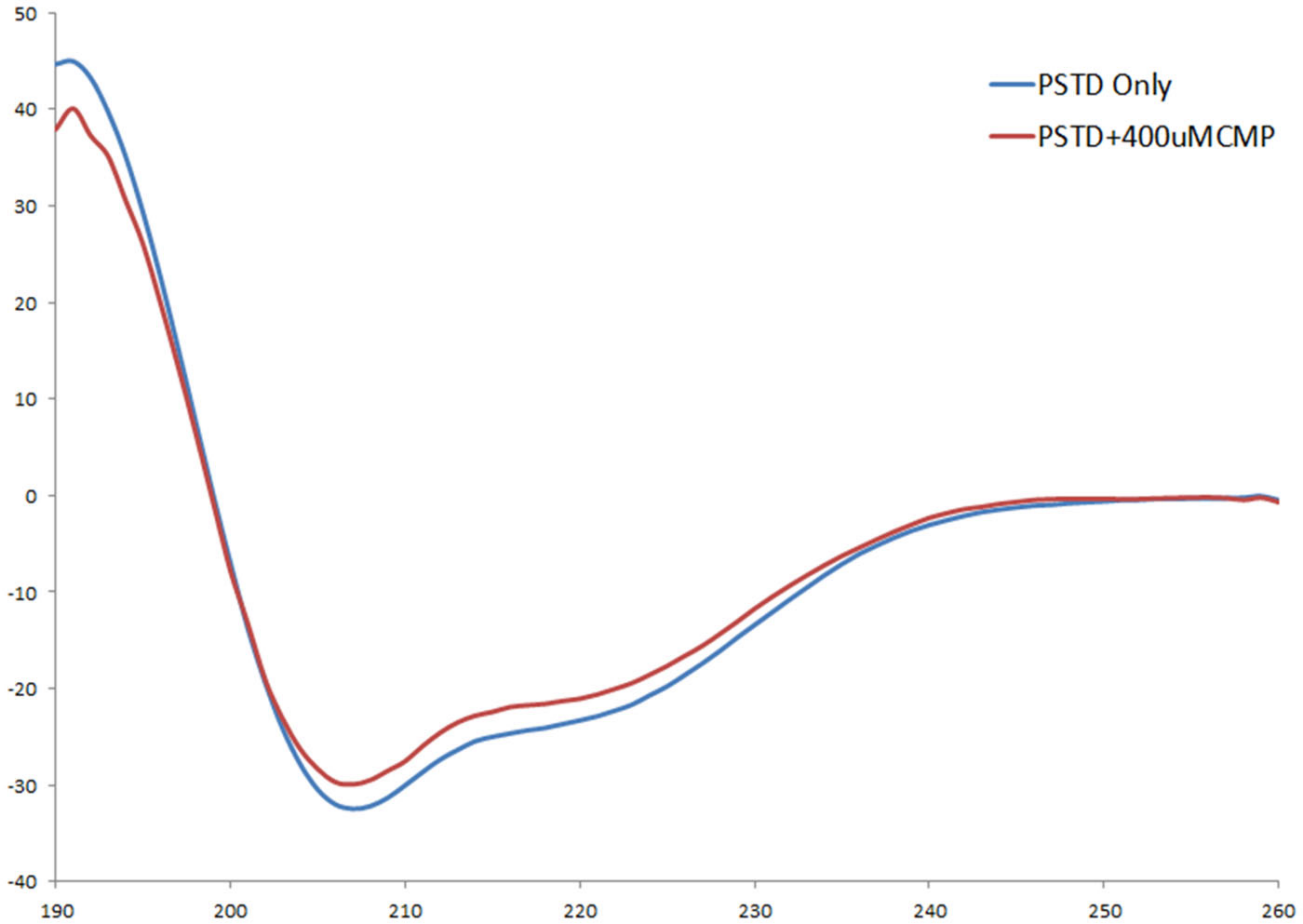
The CD spectra of the PSTD in the absence and the presence of CMP.

As shown in Figure 1, our CD spectra display that the main secondary structure of the PSTD was α-helical structure in the absence and presence of CMP ligand. The remaining half of the PSTD structure existed as a random-coil. These results are consistent with PSTD conformation in our 3-D derived ST8Sia IV molecular model^30^^,^^36^^,^^40^, and suggest the stability of the α-helical structure even in the presence of CMP.

### NMR data

Using immunoblotting and NMR spectral analyses, several inhibitors of polySia-NCAM expression have been identified^41–43^. For example, in 2013, Al-Saraireh et al. reported the pharmacological inhibition of ST8SiaII-mediated polysialylation of NCAM by CMP, and that this inhibition could modulate tumour cell migration^41^^,^^42^^,^^44^. This study demonstrated that CMP was a potential ‘druggable’ inhibitor of polysialylation, and could possibly interfere with the metastatic spread of selected polySia-NCAM positive cancers.

### The PSTD-CMP interaction

**Figure 2.**
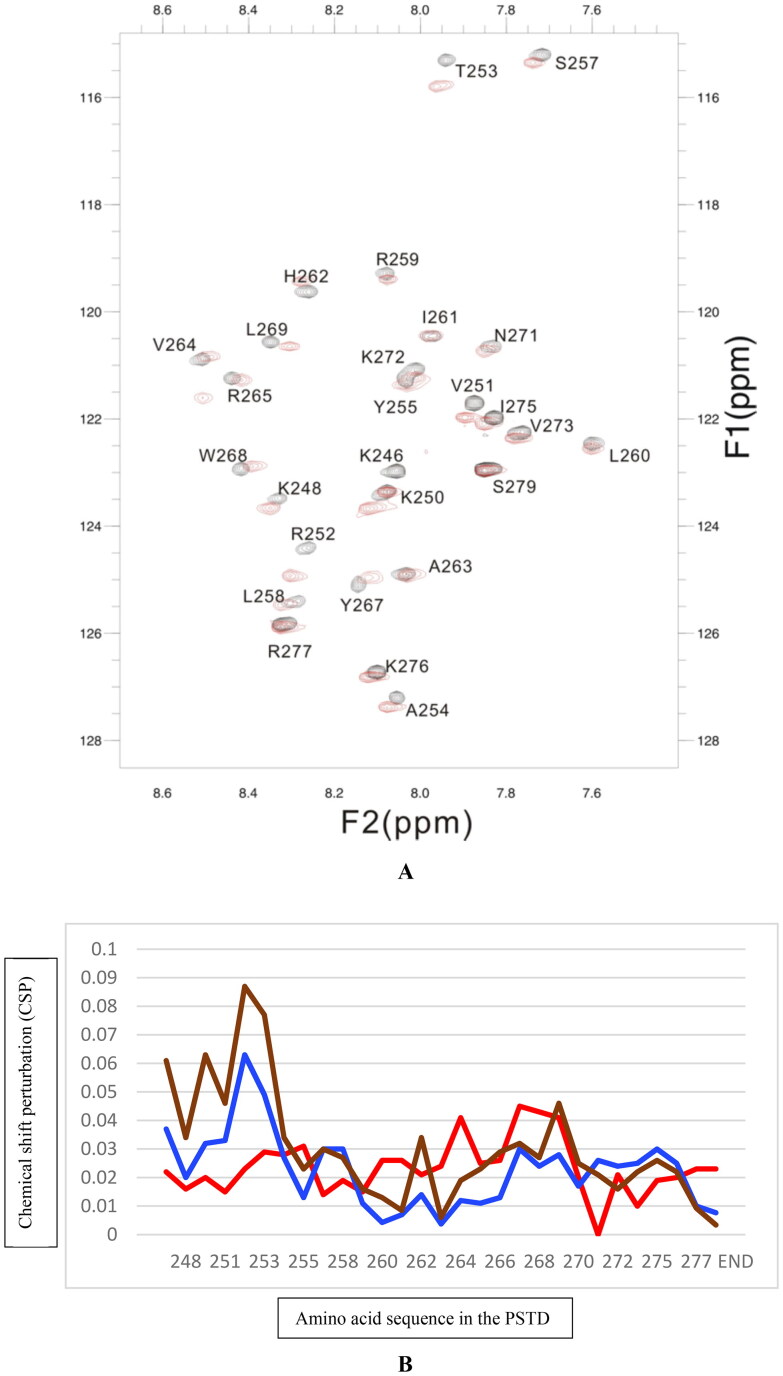
The overlaid ^1^H-^15^N-HSQC NMR spectra of the PSTD of ST8Sia IV in the absence (black) and presence (red) of CMP (A), and CSPs of the PSTD for the 2 mM PSTD - 2 mM CMP-Sia binding (blue), the 2 mM PSTD-100uM polySia binding (red), and the PSTD-1 mM CMP biding (brown), respectively (B). The CSP values ofthe (CMP-Sia) - PSTD, and the polySia - PSTD interactions are taken from the reference^30^.

**Figure 3.**
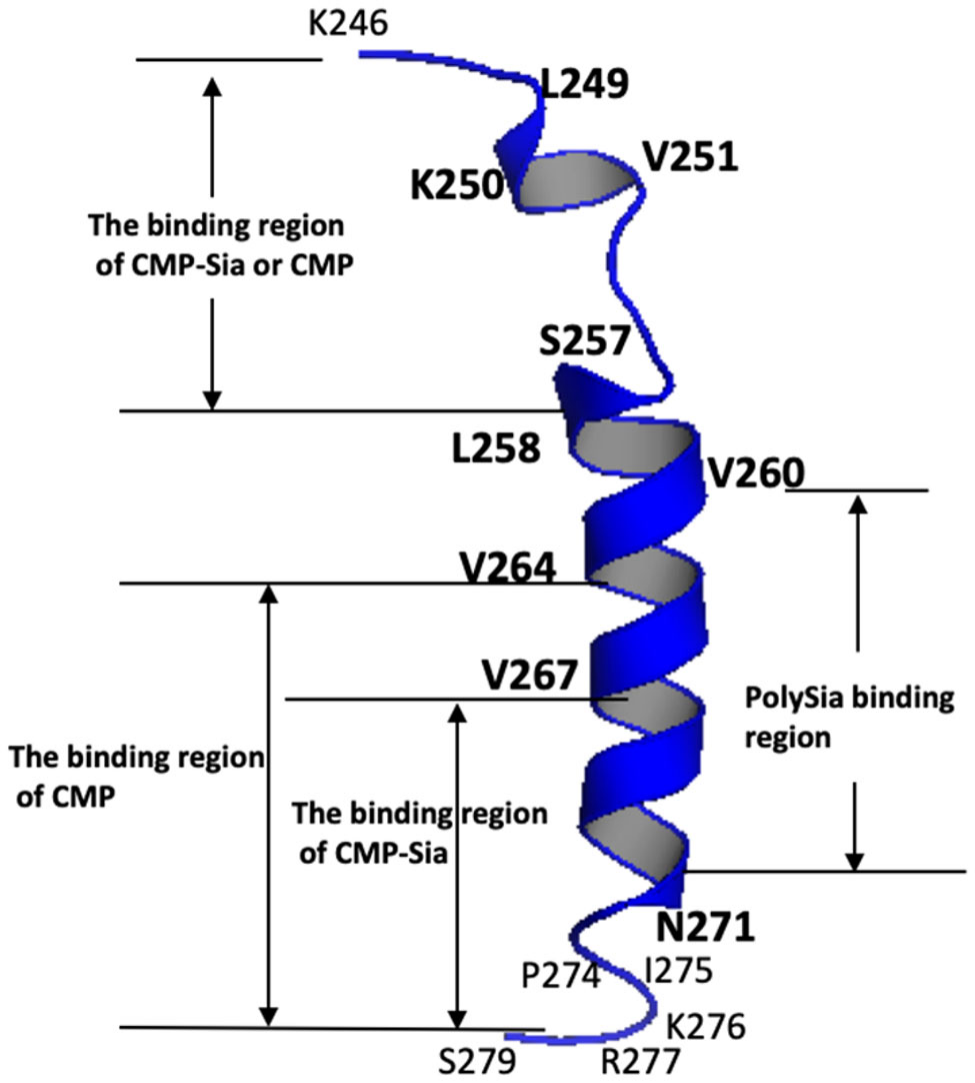
The major binding regions of CMP-Sia, polySia and CMP on the PSTD structural model based on the current and previous NMR data^30^^,^^36^.

In the present study, the binding of CMP to the PSTD in ST8Sia IV was studied by analysing 1H-15N-HSQC spectra of the PSTD in the absence and presence of CMP (Figure 2A). The binding region of CMP on the PSTD is in the residue range K246-L258, which is also CMP-Sia binding region in the PSTD (Figure 3). Another CMP binding region in the PSTD is residue range V264-K276 (Figure 3) according to the results shown in Figure 2B, in which the CSP curves for the PSTD-(CMP-Sia) and the PSTD-polySia bindings are also shown in this figure.

### Comparison in the CSPs between the CMP binding region and the CMP-Sia binding region in the PSTD

As shown in Figure 2, the largest CSPs for both the PSTD-(CMP-Sia) binding and for the PSTD-CMP binding are all focused on the residue range K246-L258, and almost all CSP values for the PSTD-CMP binding are larger than that for the PSTD-(CMP-Sia) binding in this residue range (Figure 2B). In addition, other CMP and CMP-Sia binding regions in the PSTD are residue ranges V264-K276 and V267-K276, respectively. The CSP values of most residues in the residue range V264-K276 for the PSTD-CMP binding are also larger than that for the PSTD-(CMP-Sia) binding. These results suggest that the PSTD-(CMP-Sia) binding could be inhibited by CMP.

On the other hand, there is only a polySia binding region, V260-N271, for the PSTD-polySia binding. The CSPs of all residues of the second CMP binding region (V264-K276) on the PSTD are less than that for the PSTD-polySia binding. For example, the CSPs in the PSTD from I263 to L269 for CMP-PSTD interaction are much less than that for polySia-PSTD interaction (the red curve in Figure 2(B)). Therefore, the inhibition of the PSTD-polySia binding could not be inhibited by CMP.

### Comparison in the CSPs between the PSTD-CMP interaction and the PSTD-polySia/CMP-Sia mixture interaction

In order to further analyse CMP’s inhibition effect on the polysialylation, the CSPs for the PSTD-CMP interaction were compared with the CSPs for the PSTD-polySia/CMP-Sia mixture interaction.

**Figure 4.**
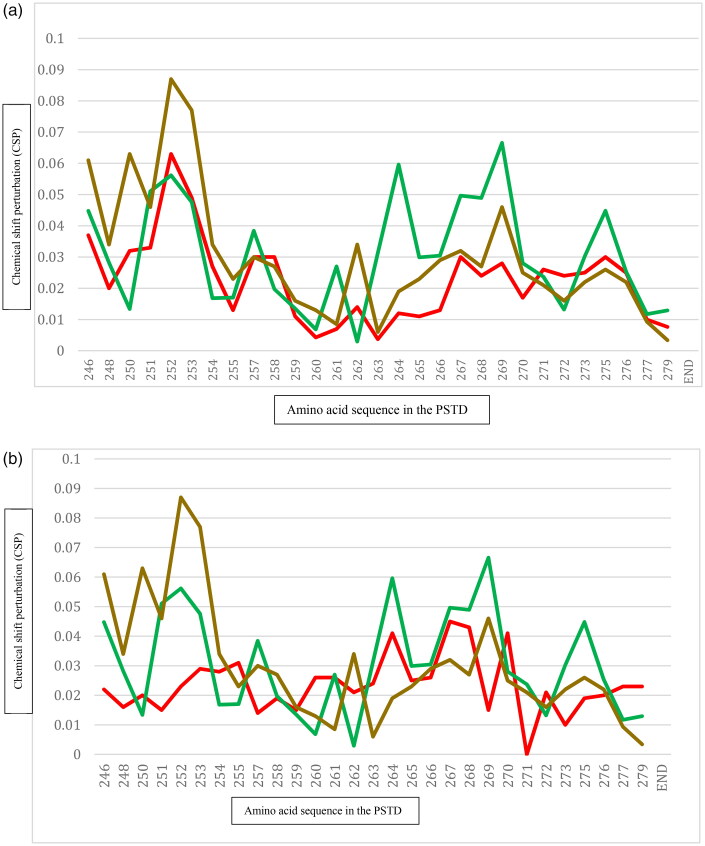
The comparison in CSPs of the PSTD for the PSTD - (CMP-Sia) binding (red), the PSTD- CMP biding (brown), and for the mixture of polySia and CMP-Sia (green) (A); The comparison in CSPs of the PSTD for the PSTD - polySia binding (red), the PSTD - CMP biding (brown), and for the mixture of polySia and CMP - Sia (green) (B). The CSP values of the all interactions are taken from references^30^^,^^36^.

It has been proposed that the non-competitive binding occurs when CMP-Sia and polySia coexist in the solution system^36^. As shown in Table 1, CMP-Sia was mainly bound to the residual range from K246-L258, but polySia was bound to the H2 helix, and the significant change in chemical shift was found at four residues V264, Y267, W268, and L269^30^^,^^36^. As shown in Figure 4, the CSPs of the PSTD for the PSTD-CMP interaction are significantly larger than that for the PSTD-polySia/CMP-Sia mixture interaction in the range K246-L258 (CMP-Sia binding region in the PSTD) (Figure 4A), but smaller than that in the range V260-N271 (polySia binding region in the PSTD) (Figure 4B). These results further suggest that the CMP-Sia binding region in the polySia/CMP-Sia mixture could be occupied by CMP only.

**Table 1.** The major NMR features of the PSTD binding to different ligands or mixture (polySia, CMP-Sia, CMP, triSia, and the mixture of polySia and CMP-Sia) based on analysis of the overlaid 1H-15N-HSQC spectra and chemical shift perturbations of the PSTD residues.

The ligand interacted with PSTD	The residual range that displayed main changes in chemical shift	The largest CSP values displayed in the following residual range	The residues displayed significant decrease in peak intensity
polySia (DP92)	R252-P256, L260-T270 (covered the whole long helix H2), K276-S279	0.045 in A263-T270. V264, Y267, and W268 have the largest change in chemical shift.	20 residues: K246, K248, K250, R252, T253, A254, Y255, S257, L258, R259, I261, H262, A263, V264, R265, Y267, W268, L269, N271, K272.
CMP-Sia	K246-A254 (covered the whole short helix H1); P256-L258, Y267-L269, N271-K276	0.063 in K246-A254; 0.030 in Y267-K276.	No
Mixture of polySia and CMP-Sia	K246-A254 (covered the whole short helix H1); A263-T270.	0.056 in K246-254; 0.067 in A263-T270. V264, Y267, and W268 have the largest change in chemical shift.	9 residues in H2 helix domain: S257, L258, R259, I261, R265, Y267, W268, L269, N271.
CMP	K246-S257(covered the whole short helix H1), V264-N271 (covered the half of the long helix H2), V273-K276	0.088 in K246-Y255; 0.046 in V264-K276	No
triSia (DP3, trimer of α-2,8-linked sialic acid)	K246-T253, Y267-T270	0.041 in K246-T253; 0.030 in Y267-T270	No

In addition, two unexpecting findings are that the CSPs of the range I263-270 for the polySia/CMP-Sia mixture are larger than that for the PSTD-polySia binding, particularly at residues V264, R265, Y267, W268, and L269 (Figure 4B), and the peak intensities of only nine residues, S257, L258, R259, I261, R265, Y267, W268, L269, and N271 are decreased in the polySia/CMP-Sia mixture in Table 1. In contrast, 20 residues were found to be significantly decreased in peak intensity for the PSTD-polySia interaction^36^ (Table 1). These results suggest that the existence of CMP-Sia may play a role in reducing the gathering of polySia chains on the PSTD.

## Discussion

The importance of these NMR and CD studies is that they reveal for the first time the molecular mechanism underlying the basis for CMP inhibition of the polyST activity by its binding to the PSTD motif in ST8Sia IV thereby preventing polysialylation of NCAM proteins. The previous biophysical studies also establish that both CMP-Sia and polySia interact with the PSTD region within the polySTs, a motif that is an obligatory requirement for polysialylation of NCAM proteins^30^^,^^36^.

According to the previous NMR studies, there are two binding regions in the PSTD for CMP-Sia interaction^36^. One is in the residue range K246-L258, and another one is in the range V267-K276. In this study, there are also two CMP binding regions for the PSTD-CMP interaction. As shown in Figure 3, the residue range of the first CMP binding region is also K246-L258, and the residue range of the second CMP binding region is the range V264-K276, which covers the second CMP-Sia binding region (V267-K276) (Figure 3).

Our NMR studies show that amino acid residues within PSTD with the largest CSP values were in the range from V251 to T253, for the (CMP-Sia)-PSTD interaction. Obviously, the CSP values in the range K246-L258 and in H2 helix region for CMP-PSTD interaction are larger than that for (CMP-Sia)-PSTD interaction (Figure 2). These suggest that (CMP-Sia)-PSTD binding could be inhibited by CMP.

On the other hand, the largest CSP values for polySia-PSTD binding were in H2 helix (R259-T270), particularly in residues V264, Y267, W268, and L269, which are consistent with the recent wenxiang diagram analysis^45^. Because the CSP values are larger for polySia-PSTD interaction than that for the PSTD-CMP interaction, and suggest that the PSTD-polySia binding could not be inhibited by CMP.

It has been proposed that CMP-Sia in the Golgi apparatus must first interact with PSTD to initiate polysialylation of NCAM proteins. This step is required before the extended elongation of the polySia chains can take place^3^^,^^4^^,^^7–11^. Thus, the inhibitions of the PSTD-(CMP-Sia) interaction and the polySia chain formation in the early stage of the polysialylation could have occurred through CMP’s addition.

The previous NMR studies also showed that 20 residues in the PSTD displayed significant decrease in peak intensity after polySia was bound to the PSTD, suggested polySia long chain molecules have been aggregated on the 20 residues (Table 1), which are mostly located on the residual ranges K246-L258 and V260-N271^30^. However, when CMP-Sia, polySia and the PSTD are co-existed in the solution system^36^, there are only 9 residues decreased in the peak intensity. These 9 residues are located in the residue range S257-N271 (Table 1 and Figure 3). This implies that the aggregation is only occurred in the H2 helix region for the mixture of the PSTD, polySia, and CMP-Sia. Although CMP-Sia may play a role in reducing the gathering of polySia chain on the PSTD, the CSPs in the polySia binding region is still larger for the PSTD-polySia/CMP-Sia mixture interaction than that for the PSTD-polySia interaction only. Thus, our results again verified that the PSTD-(CMP-Sia) could be inhibited by CMP, which could not inhibit the PSTD-polySia binding even in mixture status of CMP-Sia, polySia, and the PSTD.

Using HPLC trace extract *in vitro* experiment, Al-Saraireh et al. showed that the polysialylation of the trimer of α-2,8-linked sialic acid (triSia) was inhibited by CMP in the presence of ST8SiaII and CMP-Neu5Ac (CMP-Sia)^42^. This result is consistent with our data based on the previous NMR spectroscopy^36^. As shown in Table 1, the CSP values of the PSTD for the PSTD-triSia interaction are much less than that for the PSTD-CMP interaction whether in the CMP-Sia binding sites or the polySia binding sites on the PSTD, and thus further confirmed that CMP should be a competitive inhibitor of ST8SiaII or ST8Sia IV. However, it should be noted that triSia (DP3, where DP refers to degree of polymerisation) belongs to oligosialic acid, and polySia used in our NMR studies is DP95. It has been designated that the DP3 ∼ 7 for oligoSia, and DP ≥8 for polySia^46^. In addition, α-2,8- and α-2,6-linked polySia exist as helical structures, which have large conformational differences compared with di- and oligoSia structures^47^. Thus, it’s possible that more residues in the H2 helix of the PSTD should be bound to polySia with the increase of polySia chain extension, and the aggregation tendency of polySia on the polyST will increase with degree of polymerisation. This is why CMP’s inhibition of polysialylation should be more efficient before formation of long chain polySia.

## Conclusion

As reported herein, our NMR analysis suggested that the polysialylation could be inhibited in the presence of CMP-Sia or triSia, and confirmed that CMP is a competitive inhibitor of polySTs. But the NCAM polysialylation could be partially inhibited by CMP when polySia and CMP-Sia co-exist in solution. As a potential inhibitor, CMP may be useful in maintaining a proper balance between the neural system health and control of hypersalivation for the life process. The current studies would benefit from a stronger focus on CAMM (Computer Assisted Molecular Modelling) methods^48^^,^^49^ for the novel drug design.
